# Tuberculosis and Diabetes in India: Stakeholder Perspectives on Health System Challenges and Opportunities for Integrated Care

**DOI:** 10.1007/s44197-021-00025-1

**Published:** 2022-01-10

**Authors:** Shaffi Fazaludeen Koya, Jinbert Lordson, Salman Khan, Binod Kumar, Chitra Grace, K. Rajasekharan Nayar, Vinod Kumar, Anand M. Pillai, Lal S. Sadasivan, A. Marthanda Pillai, Abu S. Abdullah

**Affiliations:** 1 Global Institute of Public Health, Trivandrum, Kerala India; 2 Present Address: Boston University School of Public Health, Boston, MA USA; 3 Ananthapuri Hospitals and Research Institute, Trivandrum, Kerala India; 4 Independent Public Health Consultant, Patna, Bihar India; 5 Global Health Research Center, Duke Kunshan University, Kunshan, Jiangsu China

**Keywords:** Tuberculosis, Diabetes mellitus, Comorbidity, Noncommunicable diseases, Delivery of health care, Integrated care, Tb elimination

## Abstract

**Background:**

India has a dual burden of tuberculosis (TB) and diabetes mellitus (DM). Integrated care for TB/DM is still in the early phase in the country and can be considerably enhanced by understanding and addressing the challenges identified from stakeholders’ perspectives. This study explored the challenges and opportunities at individual, health system and policy level for integrated care of TB/DM comorbidities in India.

**Methods:**

We used an outlier case study approach and conducted stakeholder interviews and focus group discussions with relevant program personnel including field staff and program managers of TB and DM control programs as well as officials of partners in Indian states, Kerala and Bihar.

**Results:**

The integrated management requires strengthening the laboratory diagnosis and drug management components of the two individual programs for TB and DM. Focused training and sensitization of healthcare workers in public and private sector across all levels is essential. A district level management unit that coordinates the two vertical programs with a horizontal integration at the primary care level is the way forward. Substantial improvement in data infrastructure is essential to improve decision-making process.

**Conclusion:**

Bi-directional screening and management of TB/DM comorbidities in India requires substantial investment in human resources, infrastructure, drug availability, and data infrastructure.

## Introduction

Globally, India has the highest burden of tuberculosis (TB) and the second highest burden of diabetes mellitus (DM) [1, 2]. India had an estimated incidence of 2.64 million TB cases in 2019 of which the national program notified 2.4 million [1]. India has an estimated 75 million diabetic patients in 2021 [2]. Even with a rapid epidemiological transition in recent years [3], TB remains one of the top five causes of disability-adjusted life years (DALYS) as per global burden of diseases (GBD) data for 2019 [4]. DM triples the risk of developing TB and worsen its clinical course and outcome, whereas TB affects glycemic control in people with diabetes [5–7].

India’s Revised National TB Control Program (RNTCP), now known as National Tuberculosis Elimination Program (NTEP) evolved as one of the world’s largest public health programs that has saved at least 7.75 million lives between 1997 and 2016 [8]. Previous estimates using antibiotics sales data had shown that India’s TB program missed about a million cases annually [9], but the number of missing cases has come down drastically in recent years [3, 9]. Studies have also shown that substandard treatment for TB in India’s vast and unregulated private healthcare sector is a public health issue [10–12]. The National Program for Prevention and Control of Cancer, Diabetes, CVD and Stroke (NPCDCS) started in 2010 [13] covers the whole country since March 2016. However, estimates shows that around 39 million diabetic patients remain undiagnosed in India [2]. RNTCP and NPCDCS developed a collaborative framework in 2017, in line with the collaborative framework developed globally by WHO and the International Union Against TB and Lung Diseases in 2011 [6]. The overall goal of the program was to reduce morbidity and mortality through prevention and bi-directional screening for early detection and treatment of both the diseases. The program objective included early screening of diabetes in registered TB patients, to strengthen the referral mechanism between the programs, to strengthen the management of comorbid conditions across the programs and to establish surveillance and monitoring and evaluation mechanisms for the collaborative activities. The implementation strategies included establishing joint planning and review committees at the national, state, and district levels and establishing protocols to improve diagnosis and management through screening of all TB patients. The program aimed at improving diagnosis and management of TB among diabetes patients through intensified detection of active TB disease in healthcare settings where diabetes is managed, and by ensuring TB treatment and management. The collaboration also aims to have joint monitoring and evaluation with standardized reporting system and data sharing between the programs, joint training of program staff including those in the field, awareness and educational activities and operational research.

A recent comprehensive quantitative assessment highlights how diabetes could be emerging as the leading driver of TB incidence and mortality in India [14]. It is estimated that nearly 20% of all TB patients in India suffer from diabetes, with nine times higher odds for treatment failure, 1.6 higher odds for relapse and 1.9 higher odds for death [14].

Box: What we know already on the topic We conducted a review of literature on tuberculosis and diabetes co-management in India on four databases- PubMed, MEDLINE, Embase and Global Health. We searched the title and abstracts of publications in English, without any year restriction, using the key words ‘diabetes mellitus’, ‘tuberculosis’, and ‘India’. The literature shows that prevalence of diabetes amongst TB patients in India ranges between 12.39 and 44%. The prevalence is highest in southern states (range: 25.3% to 44%), followed by northern states (range: 12.8–15.8%). Southern state of Kerala reported the highest prevalence (44%) while central state of Madhya Pradesh reported the lowest prevalence of diabetes amongst TB patients (12.39%). Independent studies conducted in various Indian states of north, south, central, east, and western India similarly concluded that the older pulmonary TB patients (> 50 years) with overweight/obesity, alcohol users and smokers had significantly higher odds of having diabetes compared to younger patients (< 40 years). The studies noted that male TB patients had a higher prevalence of diabetes than female patients except in western India and rural India. In western India, prevalence of diabetes is more in female pulmonary TB patients whereas in rural Indian population, prevalence of diabetes is almost gender independent. Delay in seeking medical care and poor compliance with treatment contributed to the higher coexistence of TB-diabetes among the rural population. The studies also noted that pulmonary TB patients with diabetes have lower cure rate and poorer treatment outcomes compared to patients without diabetes. The studies observed that the coexistence of these two conditions increased with advanced age.

Since the diabetic patients are at higher risk of developing TB and TB worsens glycemic control of diabetic patients, the World Health Organization in 2011 recommended bi-directional screening for TB and DM. With sustained programmatic efforts, bidirectional screening is showing results. While only 29% of the notified TB patients were screened for DM in 2018, the rate increased to 64% in 2019 (2020 TB report) [1]. However, there exists wide variation across states underscoring the need for nationwide policy development to promote TB-DM bi-directional screening.

Health systems need to integrate the TB/DM services to have an effective management of these comorbidities. The effectiveness of an integrated care can be considerably enhanced by understanding and addressing the challenges identified from stakeholders’ perspectives. This exploratory study was therefore undertaken to understand the challenges and opportunities at individual, health system and policy level for integrated care of TB/DM comorbidities in India.

## Methodology

### Study Design

We used an outlier case study approach to conduct the study in two states of India: Kerala and Bihar. The south Indian state, Kerala has relatively better socioeconomic indicators and better health system, while the eastern Indian state of Bihar has relatively poor socioeconomic indicators and comparatively weak health system. To illustrate, Bihar had a TB case examination rate of 438/100,000 population while Kerala had a rate of 1,466/100,000 [1]. At the same time, Kerala (65.76/100,000) and Bihar (67.03/100,000) had similar case notification rates [15]. This shows that even with higher testing rate, Kerala reports lower TB incidence while Bihar’s low TB rates are partially attributable to the low testing rate.

We conducted exploratory in-depth interviews (IDIs) and focus group discussions (FGDs) with relevant program personnel including, field staff and program managers of TB and DM control programs and officials of partner organizations. The key topics and questions covered in the interviews are listed in Table 1.

**Table 1:** Interview questions

Topic	Main question	Follow-up question	Probe question
Perception	What do you know about the problem of TB and Diabetes coming together?	How big is the problem?	–
Case finding	What is the case finding strategy?	How are presumptive TB and DM cases detected?	What are the anticipated barriers in case detection?
Confirmation	What are the steps involved in diagnosing a suspected TB and DM cases?	How could we facilitate early diagnosis of suspected cases?	Who is responsible and what is their role?
Treatment	What are the treatment delivery methods for the integrated management?	How is the medication administered? What system is in place to ensure medicine availability?	What are the barriers in treatment adherence?
Prevention	What are the activities that are being done to prevent TB-DM in your area?	How effective are our prevention strategies? What are their strengths and weaknesses?	What can be done to prevent the worsening of TB-DM epidemic?
Challenges	What are the main challenges/barriers?	How can we overcome these challenges?	Are healthcare workers and programs getting enough support for your work? Do staff require more training to do this job well?
Suggestions	Can you suggest what more can be done?	–	–

Interview participants were identified based on their role in the TB and DM control programs or from among the officials of partner organizations working in this field (Table 2). Participants were informed about the study objectives and once they agreed to participate, a mutually convenient time was selected for the interview. Interviews were conducted either face to face or using Zoom platform. Verbal consent was obtained from all participants. Ethical approval with a written consent waiver was obtained from the institutional review board of the Ananthapuri Hospitals and Research institute, Kerala, India. The interview guide was translated to Malayalam (for Kerala) and Hindi (for Bihar).

**Table 2:** List of in-depth interviews and focus group discussions

Data collection method	Category	Number of participants
In-depth interview	Program officials	5
	Partner officials	3
	Medical officer	1
	Private clinician	1
Focus group discussion (FGD)	Healthcare workers	6 per groups, 2 FGDs
	TB program staff	6 per group, 2 FGDs

### Data Analysis

We followed the thematic analysis approach outlined by Braun and Clark for qualitative data analysis [16]. This approach has six-steps starting with getting familiarized with the data, followed by assigning initial codes, generating themes, checking validity and reliability of the themes, adding definition of the themes, and finally interpretation and reporting. The interviews and FGDs were audio recorded for face-to-face interviews or video recorded for zoom interviews. The interviews and FGDs were transcribed and translated to English and the content was analyzed manually. All transcripts were repeatedly read, and key themes were identified. We used a deductive approach to analyze the qualitative data, given the nature of the research questions and the prior knowledge (literature-based) regarding the key factors that affect integrated care. The information was triangulated through findings from different stakeholders.

## Results

Seven key themes emerged from the analysis (Table 3), which are described below.

**Table 3:** Key themes that emerged from the analysis

1. Perception of the TB/DM comorbidity as a public health problem	
2. Public sector ownership and decentralized care	
3. Private sector participation	
4. TB/DM case detection	
5. TB/DM case medical management	
6. Human resource needs	
7. Training needs	

### Perception of the TB/DM Comorbidity as a Public Health Problem

The respondents from the two states differed in their perception of the seriousness of the problem as well as their assessment of the health system’s ability to manage TB/DM. Respondents from the state with a weaker healthcare system and high TB burden (Bihar) marked a lower rating of the seriousness of the problem of TB/DM than the respondents from the state with a better healthcare system and low TB burden (Kerala). The capacity of the health system was rated poor by the respondents from Bihar compared to respondents from Kerala.

“Diabetes mellitus complicates diagnosis as well as treatment and management of TB in the state…”

(IDI, partner organization official)

“We have up to 40% prevalence of diabetes among TB patients… It can be termed “diabetic driven TB epidemic”.

(IDI, partner organization official)

“We do not know much about it (TB/Diabetes comorbidity). But we know that as cases of diabetes is increasing in our locality, out TB patients can have diabetes”

(FGD, healthcare workers, Bihar)

“A committee was formed and had met once, but there was no follow-up. Other departments are not serious with TB patients.”

(IDI, program official, Bihar)

### Public Sector Ownership and Decentralized Care

Study participants in Kerala alluded to the local government system’s role in identifying and addressing social determinants of TB. The local governments show political commitment and allocate sufficient resources to provide patient support like food rations, transportation cost, etc. The TB program staff found the local governments to be very supportive. In Bihar, district officials, NGO partners, and field staff of the TB program are concerned about the lack of political commitment as well as resources for the TB program.

“If a patient is not able to be followed-up, we do take support from local government officials like panchayat (village government) members and police officers for counseling them and to help the patient to restart the treatment regimen”

(FGD, TB program staff, Kerala)

“If government systems worked better, we would not have been here. Government system in the state (Bihar) is not working well, they are unaware of what is needed in TB control…also for TB/DM control.”

(IDI, partner organization official)

### Private Sector Participation

Kerala brought in private sector providers very early in the TB program implementation through training and engaging professional organizations like Indian Medical Association through the Global Fund supported public private mix projects. This was later continued by other projects like JEET [17]. The specialty doctors are also well informed about the criteria for finding TB cases as well as TB/DM comorbid conditions. Private sector practitioners in Kerala screen most of the TB patients for DM, although, screening of diabetic patients for TB is still lagging. However, the clinicians in the private health sector in Kerala are not so much aware and sensitized about the importance of DM as a risk factor of TB. Therefore, there are not enough efforts in the private sector for bi-directional screening of TB/DM.

“Even though TB programs are implemented through private sector, these institutions do not have exclusive staff for TB reporting and follow-ups, which should be considered as one of the weaknesses. All diabetes cases are not screened for TB in (private) institutions….””

(IDI, private provider, Kerala)

However, this “weakness” could be addressed by a close monitoring of the implementation of the STEPS initiative under the under the JEET project [17], which is a single window for notification, linkage for public health actions and treatment adherence support in every private hospital. But primary care level health workers and field staff were not aware of STEPS program.

“No, we have never heard of that (STEPS). We know TB treatment is free in government facilities. Nobody in the village is aware that TB medication and treatment is free even if they go to a private hospital”

(FGD, healthcare workers, Kerala)

On the other hand, there exist wide gaps in the engagement of private practitioners in TB control efforts including managing comorbidities in Bihar. The state could not gather enough private provider support for many years, and only recently the State started to make some progress through the Patient Provider Support Agency (PPSA) project. Even though the support was not as forthcoming as anticipated, the new intervention helped to improve case notification from private sector.

“This interface agency project has a huge role…, when we started this program as a pilot in 2014 private sector notification used to be around 50 cases annually, but in 1 year we took the notification to 18,000... government alone cannot do this.”

(IDI, partner organization official)

“We will take much time to reach uniform and standardized quality treatment in public sector, so it is better that we engage in respectful partnership with the private sector.”

(IDI, Program official, Bihar)

### TB/DM Case Detection

At present, there is a clear policy and operational framework for the management of co-morbidity of TB-DM like TB-HIV. Diabetic patients visiting the primary health centers (PHCs) and community health centers (CHCs) are directed to the NCD clinic. According to the protocol, the patients in the NCD program are first referred for counseling, where they are informed about the diabetic medications and lifestyle/diet practices. Along with this, patients are assessed for TB symptoms, and symptomatic patients are referred to the nearest TB diagnostic centers. In TB diagnostic facilities, these patients are referred to CBNAAT examination since they come under the category of vulnerable population due to comorbidity. Similarly, when a patient is first diagnosed with TB, he/she undergoes diagnosis for other comorbidities including DM. Eventually diabetic patients are identified from the TB program and TB patients are picked up from NCD clinics. Though these guidelines and standards exist, the service delivery vary between the states.

“All patients visiting NCD clinics are asked for four symptoms of TB (i.e., fever, night sweat, cough and weight loss) ...and a ‘4S’ stamp/ seal is placed. This system is a documented evidence that health workers have asked the question for the symptoms of TB to an NCD patient”.

(IDI, program official, Kerala)

In Bihar, program management of TB/DM is in the very nascent stage. There is a consensus among all respondents that much needs to be done in improving the bi-directional screening. Shortage of laboratories and inadequate testing facilities are challenges.

“Focus on testing for diabetes has increased, earlier it was not compulsory but now government has made it compulsory, but it’s not integrated even now.”

(IDI, partner organization official)

“Government says we are doing enough (diabetes) tests, but you know what is happening in PHCs. There is no specific program for control of diabetes. This is a real problem”

(IDI, program official, Bihar)

### TB/DM Medical Management

In Kerala, during the whole duration of TB treatment, the patients are monitored for both TB and DM by the TB program in coordination with the field level primary healthcare staff like junior public health nurses and junior health inspectors. The blood sugar values are documented in ‘TB patient cards’ and, patients are kept under surveillance even after TB is cured because he/she will still be vulnerable to TB relapse. The medicine supply for DM is streamlined and decentralized to a large extent.

In Bihar, TB patients requiring DM medication are most often referred to a specialist doctor. The care is not decentralized and integrated, and patients are asked to follow-up with the NCD clinics.

“Hospital can do a lot, but management in the hospital needs to be improved.”

(FGD, healthcare workers, Bihar)

“TB has separate system for medicine supply and Diabetes has separate system, they are not integrated.”

(IDI, partner organization official)

### Human Resource Needs

TB program is well integrated into the general health system in Kerala, and the existing permanent staff in the general health system consider TB as part of their job portfolio, though contractual positions under NTEP still exist. On the contrary, most of the TB program staff in Bihar are contractual positions and most positions remain vacant. Lack of staff in hospitals is also a barrier in TB case detection and testing.

“Primary health centers are still struggling to follow up TB patients due to lack of lab technicians and field supervisors. Accredited social health activists (ASHAs) are key health workers to find suspected TB cases and they help patients to come to the hospitals for the sputum testing and follow up with them till completion of treatment. But due to shortage of TB treatment supervisors and TB laboratory supervisors, the timely follow-up with presumptive cases gets delayed.”

(IDI, medical officer, Bihar)

Same is the case with DM testing and follow-up.

“Whatever human resources are available for TB care should be fully devoted to TB management. But they are assigned with many other duties and therefore we cannot optimize TB case detection and management.”

(IDI, program official, Bihar)

“Health care facilities face manpower shortage. There is no doctor and the front level staff are stretched. As there is no sufficient sputum microscopy or GeneXpert facilities, how will diagnosis happen? As a government official, I can’t say much. But if you go to the field, then you will see it yourself.”

(IDI, program official, Bihar)

### Training Needs

To improve diagnosis and treatment of TB and DM, doctors and other health workers need better training. If a TB patient is diagnosed with DM, the patient is referred to another hospital for antidiabetic treatment. Lack of adequate training for doctors and the low competence level of doctors at PHC hinder integrated management. In-service training programs for doctors and including chapters on co-management of diseases in textbooks, as part of medical curriculum or specialist training, and developing professional guidelines for TB/DM co-management will build capacity among the healthcare professionals and support decentralized care.

“Lack of confidence among doctors is an issue. In TB we know it is a standardized regimen but for diabetes it is not that standardized. So, doctors at the primary care level are not confident to manage TB/DM”

(IDI, medical officer, Bihar)

“We should have included training of doctors on TB and diabetes in the TB training module at least 5 years ago so that we could have a well-equipped system for TB –Diabetes management”

(IDI, program official, Bihar)

## Discussion

Our study adds to the existing knowledge on TB/DM integrated management in India and highlights the challenges for a seamless integration, using an outlier case study approach. There are six key messages that emerge from our study (Table 4).

**Table 4:** Key messages on improved TB/DM integrated management

1. Stronger political commitment to strengthening individual programs	
2. Effective integration with general health system through better horizontal-vertical strategy	
3. District level TB/DM program coordination	
4. Closing the training and skills gap	
5. Resource mobilization	
6. Strengthening data systems	

First, strengthening the individual programs for TB and DM is critical for integrated management of comorbidities. Kerala had a relatively strong TB control program built on a well-organized and functional health system at the primary care level, which helped to effectively implement the DOTS strategy when it was launched in the late 1990s [18], but in Bihar the TB program is still in a phase of stabilization [19]. A strong political commitment at the state level along with political stewardship at the Local Self Government (LSG) level helped Kerala to overcome many structural and financial barriers that affected other states [20]. The participation of the private healthcare sector in the state and the resultant synergy of public and private sectors helped to implement the DOTS strategy at a wider scale, compared to other states [21]. Whereas, experience from Bihar shows the scope of improvement regarding political commitment. Districts in Bihar lack basic facilities like an adequate number of functional sputum microscopy centers and X-ray machines. Shortage of clinical staff and field level program staff are the results of a long history of lack of political and administrative commitment. Organized and systematic engagement of the private sector is only a recent phenomenon in Bihar and though drug-resistant TB services and TB/HIV have been in place for a few years TB/DM services have only started recently [22].

Second, effective integration with general health systems calls for improved vertical-horizontal synergy. Absence of a designated cadre of staff for DM or NCDs at the field level and designated district level officials limits the scope of integration of programmatic management. TB has an appropriately designed vertical program in place, while DM is largely managed through the ‘horizontal’ general healthcare system. When HIV/TB integrated services were launched, there was a relative advantage as both diseases had some form of vertical structures. This is not the case with TB/ DM. DM is managed through the general health staff and many states do not have a designated district level official to manage the NCD programs. However, for many states the district nodal officer for TB and HIV is the same, (usually the district TB officer) which makes it easy to coordinate the program staff in TB and HIV programs. Adding field level staff for vertical management of NCDs may not be immediately feasible given the financial constraints. However, having a dedicated NCD official at the district level is the minimum essential requirement to coordinate the TB and DM management. Research shows that TB patients newly diagnosed with diabetes are “lost” to the health system after their TB treatment is completed [23]. Even when policy is present, poor quality of care for DM patients along with NCD services with poor monitoring and evaluation were issues in TB-DM co-management [24].

Third, coordination between the TB and DM program at the district level is essential to ensure service delivery. The national, and to a large extent the state coordination mechanisms work at the policy and supervisory level, while the district coordination is critical in ensuring clinical care and streamlining programmatic management [25]. A well-functioning district level coordination not only addresses programmatic challenges, but also streamlines clinical care. Given the complexities around TB/DM especially with the increasing number of drug-resistant TB cases, comorbid TB patients cannot be managed solely by TB program staff and district TB officers. This requires a higher-level commitment from the state leadership of both programs.

Fourth, integrated management calls for addressing training and skill gaps at multiple levels. TB and DM are complex diseases by themselves that demand expertise to manage as individual medical conditions, and this calls for additional efforts for training healthcare workers [26]. This is more relevant in the Indian context due to three reasons. One, there is considerable diversity in the availability and quality of healthcare providers across Indian states. Two, TB drug resistance is not just a programmatic challenge but also a clinical challenge in India with the scarcity of specialist physicians in the public health system in many states. Third, TB/DM comorbidity is less understood as a public health problem not only by the primary care and community level healthcare workforce, but also by specialist physicians in the private sector. The interviews revealed the apparent lack of understanding of the seriousness among healthcare workers regarding the double burden of diseases in Bihar that has a relatively poor health system capacity in addressing the challenges. Even in Kerala with a better coordination in place, private sector clinicians-both respiratory medicine specialists and diabetologists/ general physicians-need focused training on improved bi-directional screening as our study shows.

Fifth, additional resource mobilization is imperative for effective bi-directional screening. The literature suggests that it would be beneficial to integrate a symptom-based approach in routine health care to enable bidirectional screening [27]. The marginal costs for the same will also be less as compared to stand-alone screening programs [28]. Enabling factors for bi-directional screening include availability of facilities at the screening center, sensitized and trained staff, adequate financial resources for the health care facility for doing bi-directional screening and a robust NCD program. Review of studies shows that TB patients registered at tertiary and secondary health centers were more likely to be screened than primary health centers probably due to better availability of laboratories, testing facilities, positive attitude, better sensitization and awareness among treating physicians and healthcare workers [29, 30]. Studies also show that the bi-directional screening is highly acceptable in the community if the health system can mobilize required additional resources [31]. In higher level medical centers, additional resources are not required, however, healthcare providers need to be sensitized regarding the importance of bi-directional screening [32, 33].

Finally, better decision-making requires better data. There exist data gaps that impede understanding of the problem of TB/DM in India, but the gaps are not uniform across the country. The literature review shows that TB/DM research is mostly concentrated in the southern states of India, probably due to the presence of more TB research institutions that undertake field studies. It can also be due to the differences in the epidemiology of NCDs between regions. However, with increasing incidence of DM in all states across the country, research institutions and the TB and NCD program should undertake more state specific population-based and hospital-based studies. Available research are mostly epidemiological studies quantifying the burden of the problem and the treatment outcomes. Modelling TB outcomes in the event of DM comorbidity suggests that lives may be saved by diagnosis and treatment of TB among DM patients [34].

## Conclusions

The TB/DM integrated management is still evolving in India and there are challenges and opportunities. The vast network of public health facilities offers opportunities to improve the service delivery. The challenge lies in strengthening the individual programs and to envision and implement a horizontal-vertical integration. Understanding the stakeholder perspectives on the challenges and opportunities in effective integration is an important step in devising solutions. Our study points to the importance of decentralization of care and strengthening the district level coordination to enable bi-directional screening and management of TB/DM comorbidities. The disease control programs need to generate more data for better decision-making and close the skill gaps to improve integrated TB/DM management.

## Data Availability

The authors confirm that the data supporting the findings of this study are available within the article.

## Abbreviations

CHC: Community health centers
DALY: Disability-adjusted life year
DM: Diabetes mellitus
FGD: Focus group discussion
IDI: In-depth interviews
NCD: Noncommunicable diseases
NPCDCS: National Program for Prevention and Control of Cancer, Diabetes, CVD and Stroke
NTEP: National Tuberculosis Elimination Program
PHC: Primary health centers
RNTCP: Revised National TB Control Program
STEPS: System for TB Elimination in the Private Sector
TB: Tuberculosis
